# Meet and greet but avoid the heat: a reflection on the carbon footprint of congresses prompted by ERA2023

**DOI:** 10.1093/ckj/sfae062

**Published:** 2024-03-12

**Authors:** Sinead Stoneman, Frances Balmer, Louise Moore, Monica Fontana, Jan T Kielstein, Alexander Woywodt

**Affiliations:** Department of Nephrology, Cork University Hospital, Cork, Ireland; Sustainability Fellow, Lancashire Teaching Hospitals NHS Foundation Trust, Preston, Lancashire, UK; Department of Renal Medicine, Lancashire Teaching Hospitals NHS Foundation Trust, Preston, Lancashire, UK; European Renal Association Headquarters, Parma, Italy; Medical Clinic V, Nephrology, Rheumatology and Blood Purification, Academic Teaching Hospital Braunschweig, Braunschweig, Germany; Department of Renal Medicine, Lancashire Teaching Hospitals NHS Foundation Trust, Preston, Lancashire, UK

Earlier this year, the European Renal Association (ERA) held its 60th Congress (ERA2023) in Milan, Italy. The 4-day meeting was attended by thousands of nephrologists and allied healthcare professionals with an emphasis on face-to-face sessions but with provision to attend virtually. After the congress, a discussion ensued around the carbon footprint of ERA2023 and of medical congresses in general. Climate change has an increasing impact on global health [1–4]. Overall, healthcare accounts for 4.4% of global greenhouse gas emissions [5]. The interrelationship between climate change and healthcare also applies to nephrology [6, 7]. When it comes to a more sustainable approach to nephrology, consumables and water [8] have received most of the attention, which is perhaps not surprising. In comparison, the environmental impact of conference attendance has not generated much interest so far. Others have also emphasized the fact that most of the workforce lack formal training in assessing environmental impact [8]. Here, we provide a brief primer on the impact of climate change on nephrology, estimate the carbon footprint of ERA2023 and compare this with the environmental impact of nephrology overall. We also discuss what steps the ERA has already taken, provide a list of suggestions to further improve the situation and outline a vision for a future congress landscape in nephrology.

## CLIMATE CHANGE, HEALTHCARE AND NEPHROLOGY: COMPLEX INTERRELATIONSHIPS

The evidence that human-induced climate change exists and that it is having an increasing impact on our world is now beyond any reasonable doubt. Climate change is increasingly viewed as the biggest threat to human health worldwide [9, 10]. A case in point is 2023, with more extreme weather events than previous years and with the highest global daily average surface temperatures ever, the lowest level of Antarctic ice and largest area ever burned by wildfires in California [11]. Anthropogenic greenhouse gas emissions have now increased temperatures by an average of 1.1°C above pre-industrial levels [10]. The World Health Organization reported that in 2016, 24% of global deaths and 28% of deaths in children <5 years of age were due to environmental reasons [12]. This equates to 13.7 million preventable deaths per year [13]. Between 2030 and 2050 it is projected that an additional quarter of a million people will die annually because of climate change [14].

The Paris Climate Agreement to limit global temperature rise to 2°C this century was agreed upon by international leaders at the UN Climate Change Conference in 2015 [15]. Unless we change our habits and drastically reduce greenhouse gas emissions, it is projected that we will see median temperature increases of 3.2°C by 2100, with current emission trends projecting a 5% chance that the temperature increase will be <2°C [16]. A total of 9% of the annual carbon footprint of an adult living in Western Europe is estimated to come from personal flights, based on an average of one European flight per year and one long-haul flight every 3 years [17].

We are already seeing climate change impacts on kidney health and the provision of care. A pattern of renal injury linked to hot climate and recurrent or chronic dehydration has been described in rural populations across continents [18]. Air pollution has impacts on health such as lower serum albumin and haemoglobin levels in in-centre haemodialysis (HD) patients [19]. Inclement weather is associated with missing HD appointments: a 7-day cumulative exposure to inclement weather was associated with a rate ratio of missed HD appointments of 1.29–1.55 depending on the weather type [20]. Temperature increases are also likely to increase the incidence of urinary calculi, mainly through increased sweating and urine supersaturation [21]. The effects of chronic kidney disease of unknown aetiology, renal calculi and acute kidney injury events will likely increase in the future: an additional 3.7 billion heatwave days were recorded in 2021 when compared with the years 1986–2005 [2].

## AN ESTIMATE OF THE LIKELY CARBON FOOTPRINT OF ERA2023

The carbon footprint is an established estimate of climate change impact [17] and with greenhouse gas emissions expressed in terms of equivalent units of CO_2_ [22]. The environmental impacts of medical congresses are increasingly recognized. The carbon footprint of conferences is dominated by air travel to and from those meetings, but conference venues, hotel stays, the use of paper and single-use promotional products and food and other waste generation all have an environmental impact [23].

Others have previously attempted to estimate the carbon footprint of medical congresses: for example, the 3-day 2019 American Academy of Ophthalmology Congress was attended by >23 000 people with an estimated carbon footprint of just under 39 910 tons of CO_2_, of which 38 993 tons originated from transportation [24]. Based on calculations using myclimate.org, we estimate that the carbon footprint of air travel to Milan for ERA2023 was 5808 tons of CO_2_ (Supplementary Table S1). This methodology was based on previously reported carbon footprint estimates in other specialities [25–30].

**Figure 1:**
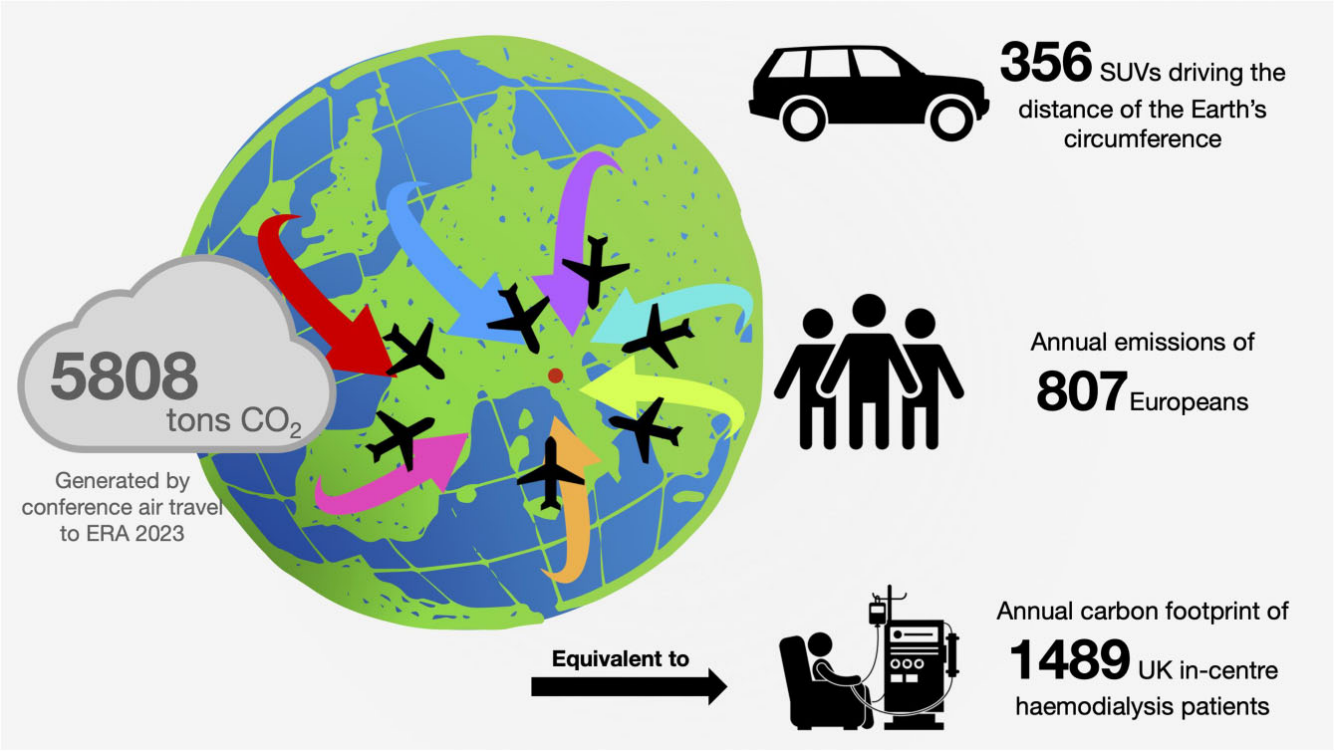
Carbon footprint of ERA2023. See supplementary tables for detailed calculations. Annual emissions of Europeans based on UNEP Emissions Gap Report 2022 [79]. Equivalent to the annual carbon footprint generated by in-centre HD for 1489 UK-based patients based on figures published by Zoccali *et al.* [80].

We also calculated the likely footprint for ERA2022 in Paris as 1691 tons (Supplementary Table 2). Given that ERA2023 had a larger in-person attendance, we think that our figures are plausible. It is also useful to put these numbers into perspective: 5808 tons of CO_2_ is equivalent to the impact of driving 356 gas-powered sport utility vehicles around the circumference of the Earth (Fig. 1). Furthermore, annual per capita emissions in Europe currently range from to 1.62 tons in Albania to 13.07 tons in Luxembourg [31]. It has been suggested that in order to have a 50% chance of maintaining a temperature increase of 1.5°C we need to have annual per capita emissions of 2.5 tons CO_2_ equivalents [32].

This estimate is an approximation and likely underestimates the true footprint, as we did not include airport transfers or stopovers and based calculations only on direct flights. Another limitation of our figures is that for ERA2023 there was a much higher number of participants where the country of origin was not documented and where we could only assume an average commute via air travel. However, our estimate is a first step in recognizing the environmental impact of ERA congresses. We encourage others to estimate the true footprint of ERA2023 or make suggestions to improve our methodology.

## WHAT DO WE KNOW ABOUT THE CARBON FOOTPRINT OF NEPHROLOGY OVERALL?

Nephrology is one of the most resource-consuming medical specialties, mainly due to the ecological footprint of HD, which is a very water- and energy-consuming treatment. About 90% of dialysis patients worldwide undergo HD. For each of the usual 156 HD treatments per year, ≈300–600 l of water are required. It is estimated that annual water consumption for all 3.5 million HD patients is 265 million m^3^. Various approaches have been described to reduce water consumption, from lowering dialysate flow [33] to using spent dialysate and other approaches [34].

When compared with HD, peritoneal dialysis (PD) has a much lower dialysate consumption of ≈10–12 l/day, although the dialysate comes in plastic bags. Production of 1000 g of plastic typically requires ≈180 l of water. Based on the weight of an empty bag of PD solution of 155 g, its production would therefore consume a volume of water that equals that required for high-volume haemodiafiltration [35]. It is important to acknowledge that waste disposal is the third major contributor to the environmental burden of dialysis affecting both PD and HD. This is further compounded by the fact that some of the disposables are considered biohazard material, which usually precludes recycling.

Consumption of electricity represents the second major problem of dialysis. About 20.7 kWh/treatment is required for HD [36]. As an example, in a Moroccan HD unit, the greenhouse gas emissions were 409.98 tons CO_2_ equivalents/year, or 5.11 tons CO_2_ equivalents/patient/year [37]. Using modern reverse osmosis technology, reducing the dialysate flow and modifying disinfection regimes can reduce consumption to <10 kWh/treatment [36].

Transport to and from dialysis and outpatient appointments represents another environmental burden [38]. Travel distances of up to 110 km to the HD facility have been reported [39]. A study from Italy described that switching five patients from in-centre HD to home HD allowed an effective reduction of 14.537 kg of CO_2_ emitted and a net economic saving of €57 975 [38].

## WHAT IS THE ERA DOING ALREADY TO ADDRESS THE CARBON FOOTPRINT OF ERA CONGRESSES?

As an organization, the ERA is conscious of sustainability issues and at its last congress it implemented a series of initiatives to mitigate this impact. These initiatives focus on travel to the conference venue, making the congress centre as environmentally friendly as possible and measures to offset the overall environmental impact of the congress (Table 1).

**Table 1:** Measures already taken by the ERA to make ERA2023 as environmentally friendly as possible

Travel to the conference venue
Encouraged all participants to use public transport
Encouraged speakers to use public transport
The conference venue itself
Air conditioning set at 26°C maximum
Avoidance of carpets throughout the conference venue (as their cleaning requires more resources and also because they need frequent replacement)
Avoidance of plastic items for catering
Complimentary refillable water bottles, avoidance of soft drinks in cans or bottles for all events directly hosted by ERA
Donation programme for leftover food
Lunch areas instead of packed lunch boxes
Delegates were required to print name badges at home; lanyards and badge holders were recyclable
Congress bags made from recyclable material available only on request
Measures to offset the carbon footprint and environmental impact of the congress
Donation to a local initiative to plant trees
Encouraged delegates to donate to this initiative as well

First, the ERA encouraged the use of public transportation and worked with the municipality of Milan to have a special train stop at the congress centre. Invited speakers were encouraged to optimize their travel. Significant efforts were also made around the venue and the congress centre itself: Air-conditioning was set at a minimum temperature of 26°C and there was no carpeting in the exhibition. Catering was another important focus and the ERA made use of local products and providers. A no-plastics policy was in operation and all ERA members could collect a refillable water bottle. Canned or bottled soft drinks were not available for ERA internal meetings and staff. To avoid waste, the staff did not get packed lunch boxes, but had a lunchroom at their disposal where environment-friendly catering was provided. There was a donation program for leftover food to avoid waste.

Further efforts were made around paraphernalia typically seen during congresses. Participants were asked to print badges at home for use with recyclable lanyards and paper badge holders. Congress bags were available only upon request and made from recyclable materials. Transport was avoided wherever possible. For example, when the ERA's standard audio visual provider from Germany needed equipment, this was rented locally and not transported from Germany.

In a conscious effort to offset some of the carbon footprint of the congress, the ERA embarked on a collaboration with Foresta.mi, an initiative of the City of Milan to plant 3 million trees in urban areas of Milan by 2030. The ERA made a donation to Foresta.mi, ensured visibility of the project and encouraged participants to donate. Ideas are already being considered for future congresses. As an example, the scientific exhibition program may award higher priority to exhibitors that use recyclable materials for their booths. The ERA also encourages the use of public transport and the most ecological transport method to reach the venue.

## A VISION: THE NEPHROLOGY CONGRESS LANDSCAPE IN 2033

**Figure 2:**
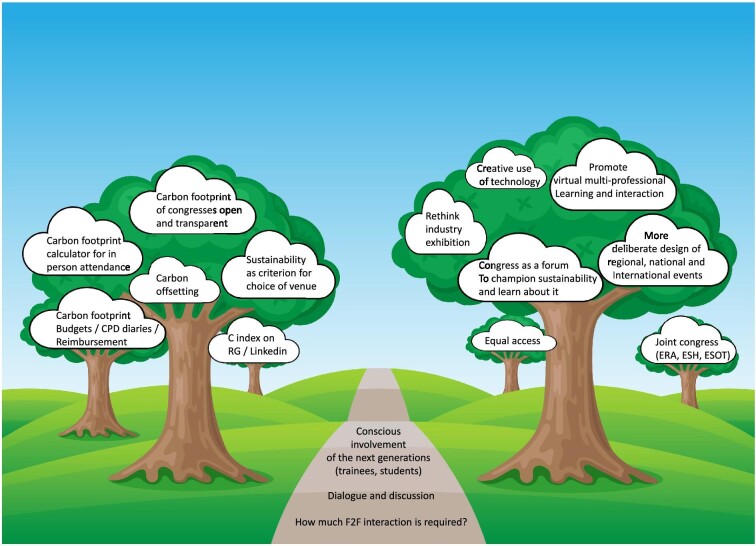
A vision of a more sustainable renal congress landscape for the future: possible actions for individuals, organizations and congress organizers. RG: ResearchGate; F2F: face-to-face. Image background modified from a Vectorstock image purchased under licence in December 2023.

The fact that action is needed to address climate change as a public health emergency is increasingly recognized [40, 41] and calls for action have been published [6, 42–51]. Somewhat surprisingly, these initiatives have not addressed conferencing. What could a more sustainable congress landscape look like (Fig. 2) and how do we get there [23]?

Carbon offsetting, i.e. compensating for CO_2_ emissions by participating in schemes designed to make equivalent reductions, appears attractive at first glance [52]. Some airlines offer the ability to add a nominal fee to your booking to offset your carbon cost of travel. In 2022 the Medical Dermatology Society Annual Meeting was attended by 284 participants with an estimated total of just under 300 000 CO_2_ equivalents caused by travel to the venue [53]. Through the use of an opt-in carbon footprint offsetting program, $485 was contributed, offsetting 16% of CO_2_ equivalent emissions generated by attendees travelling to the meeting [53]. It is increasingly recognized that carbon offsetting is not a viable long-term solution; indeed, it has been dismissed as ‘greenwashing’ by some, but could serve as an interim stepping stone to prompt behavioural change. The ERA could recommend environmentally friendly hotels and offer green travel grants to promote the use of travel through routes that are more sustainable in order to create wider awareness of the environmental impact of air travel [54].

The first step, we believe, is openness and transparency in this regard, coupled with a public commitment to make the congress the most climate friendly it can be. The ERA should be congratulated for thinking about how to improve the sustainability of ERA2023. We would urge all congress organizers, irrespective of conference size, to be equally committed as well as open and transparent. The attempts during ERA2023 should be continued with the aim of championing a more sustainable approach to congresses overall. It is vital that sustainability is considered during the early planning phase and the choice of the city and venue could be influenced by modelling its likely carbon footprint, the availability of public transport and whether and how the overall income generated during the congress is invested in sustainable projects. A dedicated sustainability team could ensure all aspects of the conference are environmentally friendly. Messaging with specific interventions for attendees to reduce their impact should feature prominently on social media and on screens during break times.

We should also do more to view congresses as an opportunity to promote ‘green nephrology’. More than 5000 delegates attended the virtual hybrid ERA-EDTA congress in 2021 during the COVID-19 pandemic [55]. The theme was ‘healthy environment, healthy kidneys’, highlighting the importance of promoting and discussing green nephrology [55]. We suggest that we should not only make the ERA congresses the most environmentally friendly they can be, but also devote a larger share of the annual congress to this topic and thereby make the ERA a visible champion of green nephrology. By doing this in combination with active measures to reduce the environmental impact and carbon footprint of medical conferences, organizations like the ERA would send a clear message about the importance of addressing climate change and potentially inspire other congresses to follow. This could cause a ripple effect within nephrology whereby participants of the congress reflect on ways they can live in a more environmentally sustainable way when they return home. Future ERA congresses could feature interactive workshops and lectures but also hands-on sessions on sustainable nephrology. Congresses could also be a great opportunity to involve our patients and their families in the discussion. A social media campaign could highlight the importance of the topic further, both to attendees and to the wider public. Our understanding is also that a sustainable nephrology award is planned for the next ERA congress in Stockholm. We also feel it may be time for one of the next congresses to feature sustainable healthcare as its flagship topic.

**Figure 3:**
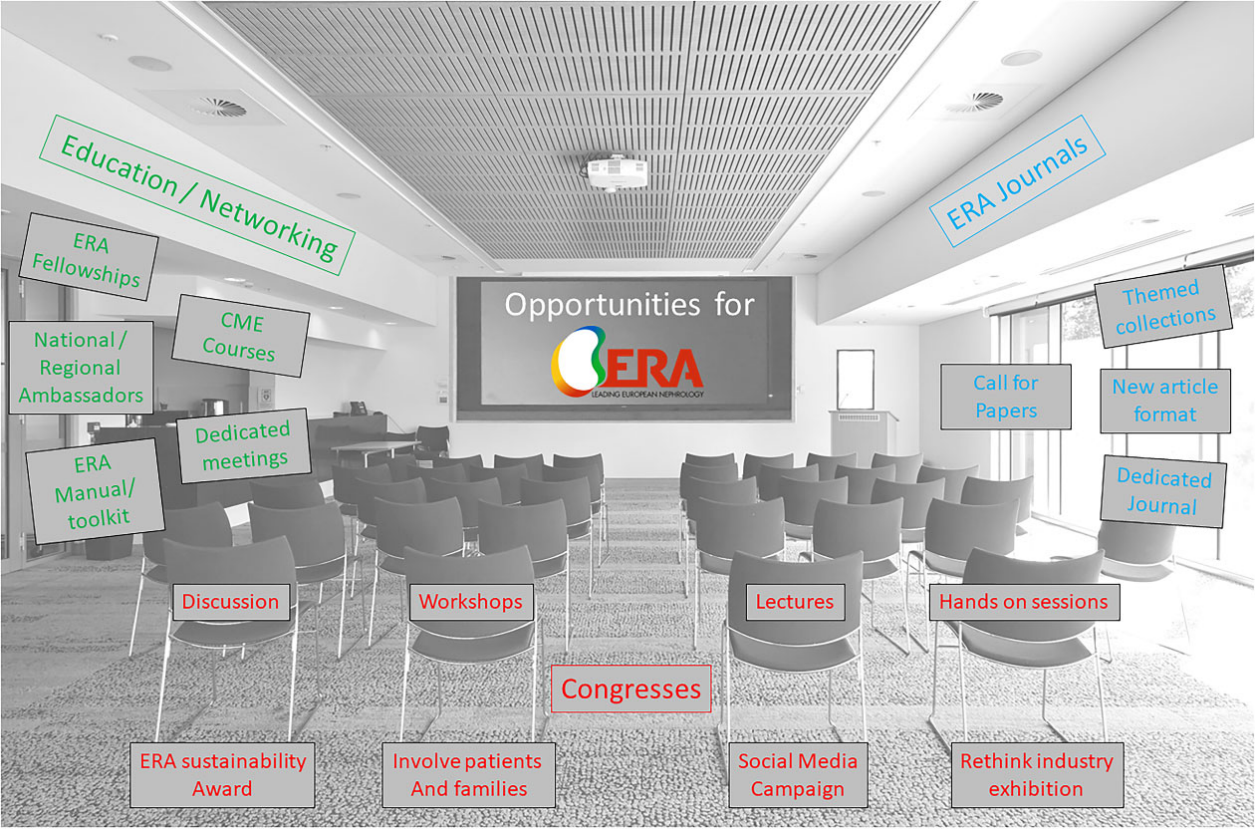
Opportunities for ERA congresses, cooperation and journals. Image background modified from a Shutterstock image purchased under licence in February 2024.

There are also wider opportunities for the ERA. In terms of its journals, ERA could consider a call for papers on this topic. A new article format, themed issues or supplements could also be envisaged. Moreover, it has not escaped our attention that there is currently no renal journal dedicated to this topic. Given the rapidly growing importance of this topic, we predict that within the next decade a journal of sustainable nephrology will come into existence and the ERA and its journals may consider this another opportunity. Education and networking are other areas where we see great opportunities for the ERA: CME courses and dedicated meetings would be relatively easy to add to the current portfolio. ERA fellowships on sustainable nephrology and local or regional ambassadors of this topic would also help to facilitate discussion. An ERA manual of sustainable nephrology is nearing publication and, together with practical toolkits, could help members access knowledge on this topic. Fig. 3 summarizes opportunities for ERA congresses and for the ERA overall.

It will be inevitable for us as individuals to make changes to our congress habits. Continuing the virtual congress option allows people to get the educational benefits of attending congresses without the environmental impact of congress attendance. For those who choose to attend in person, there could be a carbon calculator for in-person attendance at the time of booking on the ERA's website. There is also, we believe, a role for creative use of technology and virtual platforms, although even internet use has a CO_2_ footprint [56]. The COVID-19 pandemic has helped us rethink many models of care and the platforms for their delivery [57]. Nephrology congresses too had to adapt quickly to changes enforced by the global pandemic [58, 59]. In that sense, our collective experience during COVID-19 should give us some confidence and trust in the ability of medical organizations to overcome inertia to change and rapidly transition to new processes [60]. Organizations should explore technology to improve the virtual conference experience and its use for multiprofessional learning, simulation and interaction. If we were now designing medical conferences from scratch, i.e. as if they never existed in their in-person tradition, then they we would not arrive at their current format.

The climate crisis is happening now and mandates that we act. In addition to environmental benefits, virtual congresses allow participation for parents who may be unable to attend due to childcare or groups who are unable to travel due to cost. In that sense, a more conscious redesign of the conference landscape has benefits from an environmental and equity perspective. Virtual conferencing is often regarded as inferior to face-to-face conferences, but many scientists are becoming more familiar with virtual meetings [61]. The fact that the nephrology community already engages in clinical and research discussions on X (formally Twitter) [62, 63] more than other specialties demonstrates their familiarity with virtual platforms and the ability to innovate and collaborate online. Also, the industry sponsors should engage in new ways to interact with online attendees: currently organisers strive for an industry exhibition with as many square metres as possible and advertise these metrics. A new approach could be to charge industry based on a combination of square metres and sustainability indices. The wider role of industry in making nephrology more sustainable is beyond the scope of our article but is reviewed in more detail elsewhere [64].

The carbon footprint of a product refers to the cumulative CO_2_ emissions in the life cycle of a product [65]. Perhaps we should think less about individual conferences and more about the cumulative CO_2_ emissions generated over the course of an extensive career. In addition to a carbon calculator, one could consider a voluntary scheme that helps individuals keep track of their long-term carbon footprint through attending conferences. As an example, physicians in the UK keep a record of their continuing professional development (CPD) during a 5-year cycle on an educational calendar administered by the Royal Colleges. It would be easy to add a carbon footprint to such a record or to consider a CO_2_ equivalent budget for CPD [66]. On a voluntary basis, individuals could also share the carbon footprint of their career using other indices via platforms such as ResearchGate or LinkedIn. Hospitals and academic institutions could also modify their current policies of reimbursement for congress travel and reward environmentally friendly commuting. Combining congress attendance with a holiday thereafter, which is not currently possible under most reimbursement policies, could be another option to reduce air travel overall.

Lastly, collaboration will be the key to success. Now is the time for a more joined-up approach not just internationally, but also nationally and regionally. Many nephrologists in Europe attend more than one conference a year—typically the ERA, the congress of the European Society of Hypertension (ESH) and the European Society for Organ Transplantation. These will often necessitate repeated airplane travel. If national and international nephrology organizations collaborated and held joint congresses, this would result in significant reductions in CO_2_ generated by nephrologists travelling for congresses. Such a movement could lead to a ‘green’ code of conduct for all nephrology congresses and cooperation and dialogue between these major organizations and bundling of resources may lead to even more ideas in this regard. There may even be an opportunity to pave the way for an approach to conferences that other specialties may emulate later on.

## CONCLUSION

Numerous articles have highlighted the need for green nephrology [6, 42–51, 67] and many of the discussions started well over a decade ago [46, 68, 69]. Now is the time for congress travel to feature in these discussions and for us to consider our own congress habits. Here we have reflected on this topic, prompted by our experience of attending ERA2023. We welcome the steps the ERA has already taken, applaud the fact that that the ERA has established a Sustainable Nephrology Task Force [70] and welcomed the European Kidney Forum in June 2023 with its commitment to green nephrology [71]. Nephrology already accounts for substantial water consumption, greenhouse gas emission and waste [35]. Given the enormity of the challenge, all aspects of nephrology care should now be considered [72]. Many of these aspects are beyond our grasp as individual nephrologists, but changing our conference behaviour is an action that is well within our ability to control, and one that can have a very meaningful impact. We are all used to attending congresses in-person, but the evolving climate emergency [73] mandates immediate change now. We cannot allow ourselves the luxury of cognitive dissonance whereby we acknowledge climate change but regard our congress behaviour as exempt: actor Leonardo di Caprio is a famous example of cognitive dissonance when in 2016 he received an award for leadership in climate crisis and attended the ceremony in person—after flying 8000 miles in a private jet [74]. Not flying to conferences as much as we used to will be a difficult change for some of us. However, we should all note the recent 28th United Nations Climate Change conference (COP28), its acknowledgement of the link between climate change and health [75] and its pledge to transition away from fossil fuels [76, 77]. Changing our behaviour in this domain will have an immediate environmental benefit and now is the time for us all to think outside the box, avoid inertia and harness ideas from within our community and beyond. This will require a change of mindset from trying to defend the status quo towards a positive and creative rethink of the congress landscape to make it more educational, interactive and productive overall. We should also reflect on the fact that attending congresses in person is unaffordable to many colleagues in poorer countries and that it is exactly these countries that are going to face the most devastating impact of climate change [78]. A conscious redesign of the congress landscape is therefore also an opportunity for an approach that is more equitable and productive for those with fewer funds. We would like to encourage further discussion of this topic. We believe that now is the time to redesign our congress landscape and provide future generations in nephrology with opportunities to learn, collaborate and network that are equally enjoyable and productive but more conscious of the environmental impact.

## Supplementary Material

sfae062_Supplemental_Files
