# Supplementary material for: Meet and greet but avoid the heat: a reflection on the carbon footprint of congresses prompted by ERA2023
Source: Clin Kidney J. 2024 Mar 12;17(5):sfae062. doi: 10.1093/ckj/sfae062 (PMC11063956; doi:10.1093/ckj/sfae062)
Supplement: sfae062_Supplemental_Files [file sfae062_supplemental_files.zip › Supplementary Table 1.docx]

| **ERA 2023  (Milan)** | **Participants** | **Main International Airport** | **Carbon Footprint for Return  Direct Flights from Main International Airport to Milan MXP www.myclimate.org/en/** | **Carbon Footprint for Total Number of Participants** |
| --- | --- | --- | --- | --- |
| Italy | 739 |  |  |  |
| UK | 485 | London Heathrow | 0.411 | 199.335 |
| Germany | 484 | Frankfurt International | 0.292 | 141.328 |
| Spain | 425 | Madrid Adolfo Suarez-Barajas Aiport | 0.467 | 198.475 |
| USA | 414 | Atlanta Hartsfield-jackson International | 2.5 | 1035 |
| France | 350 | Paris Charles de Gaulle | 0.321 | 112.35 |
| Switzerland | 283 | Zurich Airport | 0.217 | 61.411 |
| Philippines | 259 | Ninoy Aquino International Airport | 3.5 | 906.5 |
| Netherlands | 251 | Amsterdam Airport Schiphol | 0.374 | 93.874 |
| Greece | 198 | Eleftherios Venizelos International | 0.567 | 112.266 |
| Onsite Participants   from Known Country | 3149 |  |  | 2860.539 |
| Mean Carbon Footprint per participant |  |  |  | 0.9084 |
| Onsite Participants   from Unknown Country | 3245 |  |  | 2947.745 |
| **Total** |  |  |  | 5808.284 |

**Supplementary Table 1**
