# Supplementary material for: Meet and greet but avoid the heat: a reflection on the carbon footprint of congresses prompted by ERA2023
Source: Clin Kidney J. 2024 Mar 12;17(5):sfae062. doi: 10.1093/ckj/sfae062 (PMC11063956; doi:10.1093/ckj/sfae062)
Supplement: sfae062_Supplemental_Files [file sfae062_supplemental_files.zip › Supplementary Table 2.docx]

| **ERA 2022**  **(Paris)** | **Participants** | **Main International Airport** | **Carbon Footprint for Return**  **Direct Flights from Main International Airport to Paris CDG www.myclimate.org/en/ CO2 t** | **Carbon Footprint for Total Number of Participants** |
| --- | --- | --- | --- | --- |
| France | 460 |  |  |  |
| Germany | 451 | Frankfurt International | 0.282 | 127.182 |
| UK | 374 | London Heathrow | 0.255 | 95.37 |
| Italy | 364 | Rome Fiumicino Apt | 0.454 | 165.256 |
| Spain | 348 | Madrid Adolfo Suarez-Barajas | 0.444 | 154.512 |
| USA | 317 | Atlanta Hartsfield-jackson International | 2.3 | 729.1 |
| Netherlands | 258 | Amsterdam Airport Schiphol | 0.268 | 69.144 |
| Swizerland | 218 | Zurich Airport | 0.289 | 63.002 |
| Greece | 166 | Eleftherios Venizelos International | 0.765 | 126.99 |
| Belgium | 151 | Brussels Airport | 0.229 | 34.579 |
| Onsite Participants   from Known Country | 2647 |  |  | 1565.135 |
| Mean Carbon Footprint per participant |  |  |  | 0.5913 |
| Onsite Participants   from Unknown Country | 213 |  |  | 125.943 |
| **Total** |  |  |  | 1691.079 |

**Supplementary Table 2**
